# Interferon Regulatory Factor (IRF)-1 Is a Master Regulator of the Cross Talk between Macrophages and L929 Fibrosarcoma Cells for Nitric Oxide Dependent Tumoricidal Activity

**DOI:** 10.1371/journal.pone.0117782

**Published:** 2015-02-06

**Authors:** Flavia R. F. Nascimento, Eliane A. Gomes, Momtchilo Russo, Ana P. Lepique

**Affiliations:** 1 Department of Pathology, Center of Biological Sciences and Health, Federal University of Maranhão, São Luís, Brazil; 2 Department of Imunology, Institute of Biomedical Sciences, University of São Paulo, São Paulo, Brazil; University of Nebraska - Lincoln, UNITED STATES

## Abstract

Macrophage tumoricidal activity relies, mainly, on the release of Tumor Necrosis Factor alpha (TNFα) and/or on reactive oxygen or nitrogen intermediates. In the present work, we investigated the cytotoxic activity of resident peritoneal macrophages against L929 fibrosarcoma cell line *in vitro* and *in vivo*. Resident macrophages lysed L929 cells in a mechanism independent of TNFα and cell-to-cell contact. The cytotoxic activity was largely dependent on nitric oxide (NO) release since treatment with L-NAME (NOS inhibitor) inhibited L929 cells killing. Macrophages from mice with targeted deletion of inducible NO synthase (iNOS) together with L929 cells produced less NO and displayed lower, but still significant, tumoricidal activity. Notably, NO production and tumor lysis were abolished in co-cultures with macrophages deficient in Interferon Regulatory Factor, IRF-1. Importantly, the *in vitro* findings were reproduced *in vivo* as IRF-1 deficient animals inoculated i.p with L929 cells were extremely susceptible to tumor growth and their macrophages did not produce NO, while WT mice killed L929 tumor cells and their macrophages produced high levels of NO. Our results indicate that IRF-1 is a master regulator of bi-directional interaction between macrophages and tumor cells. Overall, IRF-1 was essential for NO production by co-cultures and macrophage tumoricidal activity *in vitro* as well as for the control of tumor growth *in vivo*.

## Introduction

Activated macrophages are endowed with antitumor activities that in part are dependent on nitric oxide (NO) production [1]. Inducible NO synthase (iNOS or NOS2) is one of the enzymes that catalyses the conversion of arginine to NO and citruline. NO produced in such reactions has a role in diverse biological processes, including microbicidal activities, regulation of inflammatory and immune processes and control of tumor growth [2]. Bi-directional interactions between macrophages and tumor cells with stimulatory or inhibitory activities on NO production have been reported [3]. For instance, some tumors such as fibrosarcomas induce NO production in murine and human macrophages, whereas other tumors such as advanced melanoma or cervical cancer suppress NO production [4,5,6].

Interferons (IFNs) are key elements in iNOS expression regulation [6,7]. It is known that IFNs signaling is initiated by two distinct cell-surface receptors, type I IFN receptor (IFNAR) and the type II IFN receptor (IFNGR) [7]. Signaling through IFNAR/STAT1 leads to the formation IFNα-activated factor that mediates the activation of interferon regulatory factor 1 (IRF-1) gene by binding to IFNγ-activated sequence (GAS) in IRF-1 promoter [8]. Similarly, type II IFN signaling through IFNGR/STAT1 also results STAT1 homodimers binding to GAS and IRF-1 gene transcription [9]. IRF-1 is the first described member of the family transcription factors known as Interferon Responsive Factors [10], which have fundamental roles in responses against intracellular pathogens, including induction of iNOS and consequent NO production. IRF-1 binds to the IFN regulatory factor element (IRF-E) present in the iNOS promoter, and together with NFκB, C/EBP and STAT1, activate its transcription [11]. Using a knockout mouse model, Kamijo and colaborators showed that IRF-1 activity is essential for iNOS expression in mice [12]. Thus, IRF1 seems to be at the crossroad of type I and type II IFN signaling for NO production [12,13].

L929 murine fibrosarcoma cell line treated with Actinomycin D (ActD), a transcription inhibitor, becomes extremely susceptible to TNFα induced cytotoxicity [14,15], where TNFα treatment promotes ROS production prior to cell death [16]. However, in absence of ActD, treatment with IL-1β and IFNγ induces cytotoxicity in L929 cells, via the p38/NFκB/iNOS/NO pathway [17]. In this case, IRF-1 is an element of convergence of these pathways, once, as described before, it is central for IFN signalling, but also may be expressed upon p65 NFκB binding to its promoter. Therefore, IRF-1 links different pathways in the cells that may culminate with iNOS expression and NO production. We have previously shown that in the absence of ActD, L929 fibrosarcoma cells became sensitive to lysis by activated macrophages in an NO dependent and TNFα independent mechanism [18]. Other studies reported that cytokine or LPS activated macrophages when co-cultured with L929 cells produce high concentrations of NO [19,3] and induced tumor cell lysis in a cell-to-cell contact dependent mechanism [20,21,22]. Moreover, cisplatin treated macrophages co-cultured with L929 cells produced NO that mediated L929 cell death [23].

Most studies, including ours, showed NO dependent L929 tumoricidal activity using *in vitro* or *ex vivo* activated or stimulated macrophages. However, few studies focused on the interaction between L929 tumor cells and resident macrophages without artificial activation, a situation that is likely to occur *in vivo* upon the development of tumors. In the present work, we investigated the interaction of peritoneal resident macrophages with L929 cells *in vitro*. Moreover, we determined the host’s immune-molecular mechanisms that were triggered after *in vivo* injection of L929 tumor cells. We used macrophages obtained from mouse strains that are deficient in key regulatory molecules involved in IFN signaling or in iNOS expression. Specifically, we determined whether MyD88, iNOS and IRF-1 were relevant molecules for the control of tumor cell growth *in vitro* and *in vivo*. Our results showed that IRF-1 is a master regulator of bi-directional interaction between macrophages and tumor cells. IRF-1 was required for NO induction, macrophage tumoricidal activity *in vitro* and for the control of tumor growth *in vivo*.

## Material and Methods

### 1. Mice

Six to twelve-week-old C3H/HePas, C57BL/6, 129/SV, iNOS-/-, MyD88-/-, and IRF-1-/- female mice were bred in our animal facilities at the University of São Paulo, under standard pathogen-free conditions. iNOS-/- mice were originally from Jackson Laboratories (Bar Harbor, ME), MyD88-/- [24] mice were kindly provided by Dr. Bernard Ryffel (Centre National de la Recherche Scientifique, Orléans, France), and IRF1-/-[25] mice were kindly provided by Dr. Luiz Fernando Reis (Hospital Sirio Libanês, São Paulo, Brazil). All procedures described were reviewed and approved by the Animal Ethics Committee of the Institute of Biomedical Sciences, University of Sao Paulo, in accordance with COBEA (Brazilian College of Animal Experimentation), protocol 151/2011.

### 2. Media and reagents

RPMI 1640 culture medium was supplemented with 10 mM HEPES, 11 mM sodium bicarbonate, 2 mM L-glutamine, 100 μg/mL penicillin, 100g/mL streptomycin, 23 mM L-asparagine, 1 mM folic acid, 0.1 mM pyruvic acid and 5% fetal calf serum. This medium will be referred to as complete medium and was used to maintain all the cell cultures. All these reagents were purchased from Sigma Chemical Co., St Louis, MO, including trypsin, N-nitro-L-arginine methyl ester (L-NAME), actinomycin D (ActD), paraformaldehyde (PFA), bovine serum albumin (BSA), sodium nitrite, sulphanilamide, naphthylene diamine dihydrocloride and glycine. Rabbit polyclonal antibodies against murine TNFα and recombinant interferon-γ(IFNγ) were purchased from Endogen, Boston, MA. Sodium nitroprusside (SNP) was purchased from Riedel-DE Haen AG, Seelze-Hanover (Germany). APC (Allophycocyanin) conjugated and biotin conjugated anti-mouse CD45, clone 30-F11, and FcBlock (anti-CD16/CD32) were purchased from BD Biosciences (San Jose, CA). Biotin blocking kit, immunohistochemistry detection kit ready to use VECTASTAIN Elite ABC Reagent, 3–3’diaminobenzidine peroxidase detection reagent and Harry’s hematoxylin were purchased from Vector Laboratories (Burlingame, CA). DAF-2, 4, 5-diaminofluorescein diacetate, was purchased from Enzo Life Sciences (Farmingdale, NY).

### 3. Peritoneal cells harvesting

Mouse peritoneal cells were harvested by washing the peritoneal cavity with 5 mL sterile ice-cold PBS. Total cell numbers were estimated by counting cells in hemocytometer. Differential cell counts were determined by cytospin preparations stained with Instant-Prov (Newprov, Pinhais, Brazil).

### 4. L929 cell cultures and treatments

L929 fibrosarcoma cell line was originally obtained from the American Type Culture Collection (Rockville, Maryland, USA) and has been maintained in our laboratory in 10% fetal bovine serum RPMI 1640 medium at 37°C in humidified air containing 5% CO_2_.

L929 supernatant (L929 sup) was obtained from 48 hours cell cultures (1x10^6^/mL). After harvesting, supernatants were filtrated through 0.22 μm Millipore membrane and used in macrophage cultures diluted 1:1 (v/v) in RPMI medium.

To evaluate the direct cytotoxic effect of NO in L929 cells, the sodium nitruprusside (SNP), an NO donor, was added to L929 cells culture at a 10^–1^, 10^–2^, 10^–3^ and 10^–4^ M concentration for 48 h.

For co-culture experiments, adherent L929 cells were detached by trypsin solution treatment (0.4 g Trypsin, 8.0 g NaCL, 0.4 g KCL, 1.0 g Glucose, 0.350 g NaHCO_3_, 0.2 g EDTA in 1L H_2_O), replenished with 10 mL of complete medium and centrifuged once (150 g, 10 min, 4°C). Cell viability determined by Trypan Blue exclusion. Cells were seeded in 96-well flat-bottom microplates, 3.5x10^4^ cells/well, and incubated for 24 h until obtaining a monolayer.

### 5. Macrophage/L929 cells co-cultures

We first seeded 3.5x10^4^ L929 cells/well on flat bottom 96 wells plates and incubated them for 24 hours to generate monolayers. Resident peritoneal cells were harvested with sterile ice-cold PBS, counted and adjusted to 2x10^6^ cells/ml. These cells seeded over the L929 monolayers at a final density of 2x10^5^ cells/well (as indicated in the figure legends) and incubated for 48 hours. At the end of this period, we harvested supernatants for Griess assay, while cells were fixed and stained with Crystal Violet as described bellow. To confirm that macrophages were the agents of the effects we were observing, we also used adherent peritoneal cells from individual mice, which were detached, washed and seeded over L929 monolayers at a density of 5x10^4^ cells/well. No differences were obtained between co-cultures with total peritoneal cells and adherent peritoneal cells. In this work we will show only data regarding the total peritoneal suspension and for the sake of simplicity, heretofore we will address resident peritoneal cells as peritoneal macrophages.

In some experiments, before the macrophage addition to the plates, L929 cells were fixed with 1% paraformaldehyde (PFA) in phosphate-buffered saline (PBS) for 25 min at room temperature, followed by washing with PBS containing 1% glycin and 1% bovine serum albumin (BSA) for 30 min at 37°C. In another set of experiments, L929 cells were treated with 2 μg/mL ActD at 37°C for 1 hour, when the wells were carefully washed two times with RPMI medium.

### 6. Treatments in the co-cultures

Rabbit polyclonal antibodies to murine TNFα (20μg/mL), L-NAME (2.5, 5.0 or 10mM) and aminoguanidine (0.1 to 100 mM) were added to co-cultures of murine macrophages with L929 and were maintained throughout each experiment (48 h). Mouse recombinant IFNγ was added to the co-cultures with macrophages from IRF-1 knockouts at 2.5, 5.0 and 10 μg/mL.

### 7. Transwell assays

In some experiments, macrophages were cultured by 48 h separated from L929 cells by a cell-impermeable membrane (Transwell culture, Costar, 0.4 μm pore size).

### 8. Detection of NO production

The NO production was quantified by the accumulation of nitrite (as a stable end product) in the supernatants by the Griess reaction. Briefly, 50μL of supernatants were incubated with an equal volume of Griess reagent at room temperature for 10 min. The absorbance at 550nm was determined on a Dynatech microplate reader. Nitrite concentration was determined from a sodium nitrite standard curve.

Alternatively, to demonstrate that the macrophage and L929 cells were producing NO we used the NO-sensitive dye, 4, 5-diaminofluorescein diacetate (DAF-2) [26]. Adherent cells were incubated at 37°C with 12.5 μM DAF-2 in 0.1 M phosphate buffer (pH 7.4) containing 0.45 μM CaCl_2_. After 2 h, digital images were acquired on a Nikon E1000 microscope equipped for epifluorescence (excitation at 485 nm; emission 538 nm). The images were analyzed using the Image software (NIH, USA) by measuring the mean optical density of the fluorescence observed in the cells in relation to the background staining.

### 9. Cytotoxicity assay

L929 cell death was determined by the crystal violet staining method. After 48 h of co-culture, L929 cells were stained by adding 10 μL of a solution containing 0.5% crystal violet and 30% acetic acid to the remaining 50 μL of cell medium culture for 10 minutes. Excess stain was removed under a tap water rinse, and the plate was air-dried. A volume of 100 μL of absolute methanol was added to dissolve the stain and the absorbance was determined at 630 nm on a Dynatech microplate reader. The macrophage cytolitic activity was expressed as the percentage of tumor cytotoxicity where % cytotoxicity = (1- O.D. of L929 cells co-cultured with macrophages/ O.D. of control L929 cells) x100. Importantly, 2x10^5^ peritoneal macrophages cultured for 72 hours resulted in staining correspondent to 27% of the L929 monolayers, while co-cultures resulted in 10% due to cell death. This result indicates that macrophages are washed away before staining and do not interfere in the L929 cell viability assay (S1a Fig.)

### 10. L929 tumor cell inoculation in mice peritoneal cavity

L929 tumor cells (5x10^5^/mouse, in 100 μL sterile PBS) were inoculated intraperitoneally in mice. Cells were harvested seven days later as previously described.

### 11. Flow cytometry analysis

Aliquots of 10^6^ cells from the peritoneal cavity were transferred to 1X Hank’s Salt Solution supplemented with 15 mM HEPES pH 7.4, 5% fetal bovine serum, 0.5 U/ml DNase I. After blocking with FcBlock (antiCD16/CD32, BD Biosciences, San Jose, CA), cells were incubated with APC conjugated anti-CD45 for 20 min and washed (BD Biosciences, San Jose, CA). Cell samples were analyzed in a FACSCalibur, where we used the FL2 channel to determine cells autofluorescence and FL4 to detect cells stained with anti-CD45. We determined the absolute numbers of L929 cells in the peritoneal cavity of each mouse by multiplying the percentage of CD45- cells in each sample by the total number of cells in the peritoneal lavage.

### 12. Immunocytochemistry

Aliquots of 4x10^4^ peritoneal cells were fixed to a glass slide using a cytospin centrifuge. Cells were hydrated (3 PBS washes), endogenous peroxidase activity quenched with 0.3% H_2_O_2_ and endogenous biotin blocked with Biotin Block kit reagents (Vector Laboratories, Burlingame, CA), prior to non specific antigen blocking with 5% fetal bovine serum and 0.05 μg/mL FcBlock (anti-CD16/CD32, BD Biosciences, San Jose, CA) in PBS for 30 minutes. After aspirating the blocking solution, cells were incubated with biotinylated anti-CD45 diluted in 5% fetal bovine serum in PBS for one hour at room temperature. After washing, we incubated the cells with ready-to-use stabilized ABC reagent for 30 min, at room temperature. Antibody complex detection was made with 3’3-Diaminobenzidine (Vector Laboratories, Burlingame, CA). Cells were counterstained with Harris Hematoxylin (Vector Laboratories, Burlingame, CA) and mounted with Permount (Fisher Scientific, Loughborough, UK). Images were acquired with an Olympus BX61 fluorescence microscope (Olympus, Center Valley, PA). Number of L929 cells was estimated by multiplying the percentage of CD45- by the total number of cells in the peritoneal lavage.

### 13. Statistical analysis

All experiments were performed at least three times and one representative experiment is presented. Differences between experimental groups were tested for significance through the Student’s *t*-test, where p<0,05 indicated statistically significant results.

## Results

### 1. L929 cell lysis is dependent on NO production

**Fig 1:**
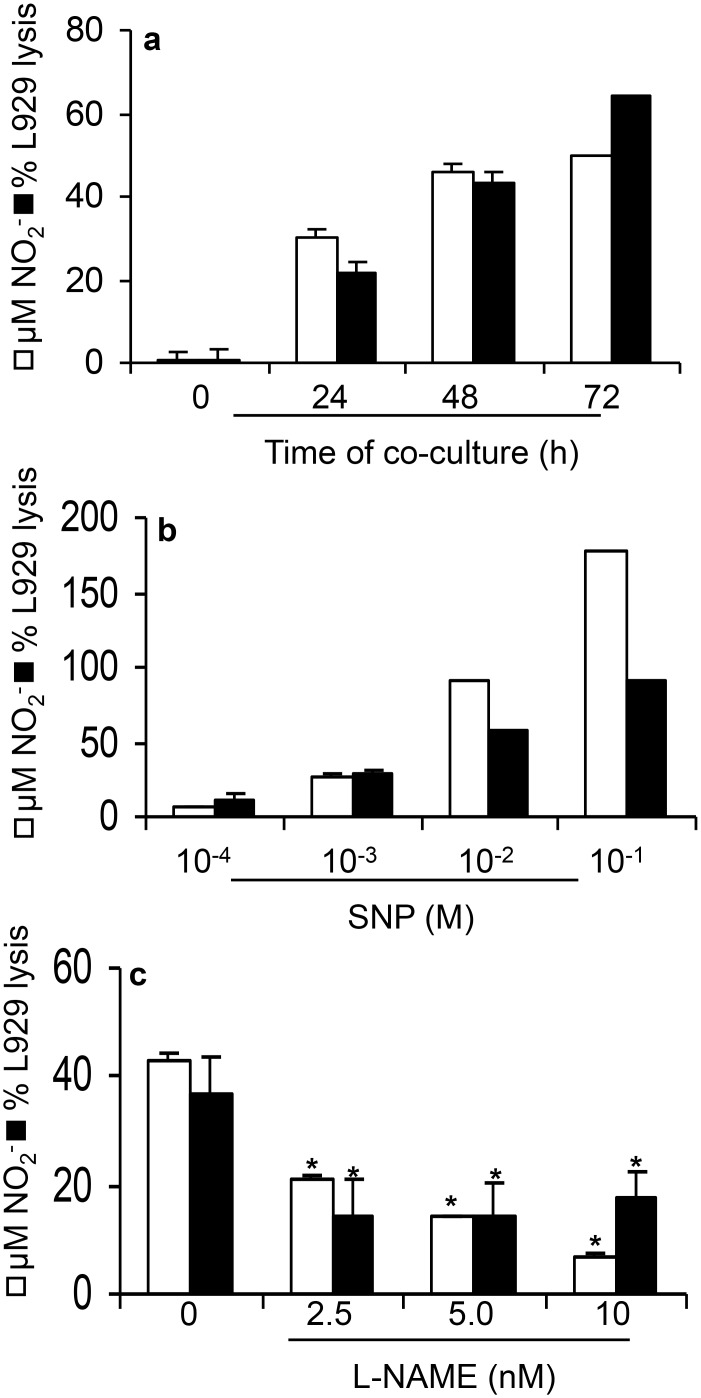
Nitric oxide production and cytotoxic activity by murine resident macrophages co-cultures with L929 cells. Mouse peritoneal macrophages **(a-c)** were co-cultured with L929 cells (2x10^5^ leukocytes: 3,5x10^4^ L929 cells). **(a)** Nitric oxide and cell viability kinetics. Co-cultures were incubated for 24, 48 or 72 h. **(b)** Co-cultures in the presence of the indicated concentrations of NO donor, SNP, for 48 hours. **(c)** Co-cultures in the presence or absence of NO inhibitor L-NAME (10mM) for 48 hours. In all cases, nitrite concentration was determined by Griess Reaction and macrophage cytotoxicity by crystal violet method. One representative experiment out of three is shown. Data represent the means ± SD of quadruplicates. Medium indicates control, untreated co-cultures.

In co-cultures of L929 fibrosarcoma cells with resident macrophages we found a significant NO production in 24h, which increased further upon 48 to 72h of culture. Paralleling NO production, we observed cytotoxicity against L929 cells (Fig. 1a). Resident macrophages or L929 cells cultured alone did not release NO (data not shown). To establish the role of NO in L929 cells lysis, we treated cultures with sodium nitroprusside (SNP), a NO donor, or L-NAME, a nitric oxide synthase (NOS) inhibitor at increasing concentrations [27]. Treatment with SNP treatment increased NO release and L929 cell lysis in a dose-dependent manner (Fig. 1b). Conversely, L-NAME inhibited NO production and L929 cell lysis in a dose-dependent manner (Fig. 1c).

Importantly, resident macrophages as well as L929 cells cultured alone did not produce detectable levels of NO (S1b Fig.), indicating that resident macrophages are not activated, confirming previous work from our laboratory, where we compared resident and BCG activated macrophages [28].

### 2. L929 cell lysis and NO production are independent of cell-to-cell contact

**Fig 2:**
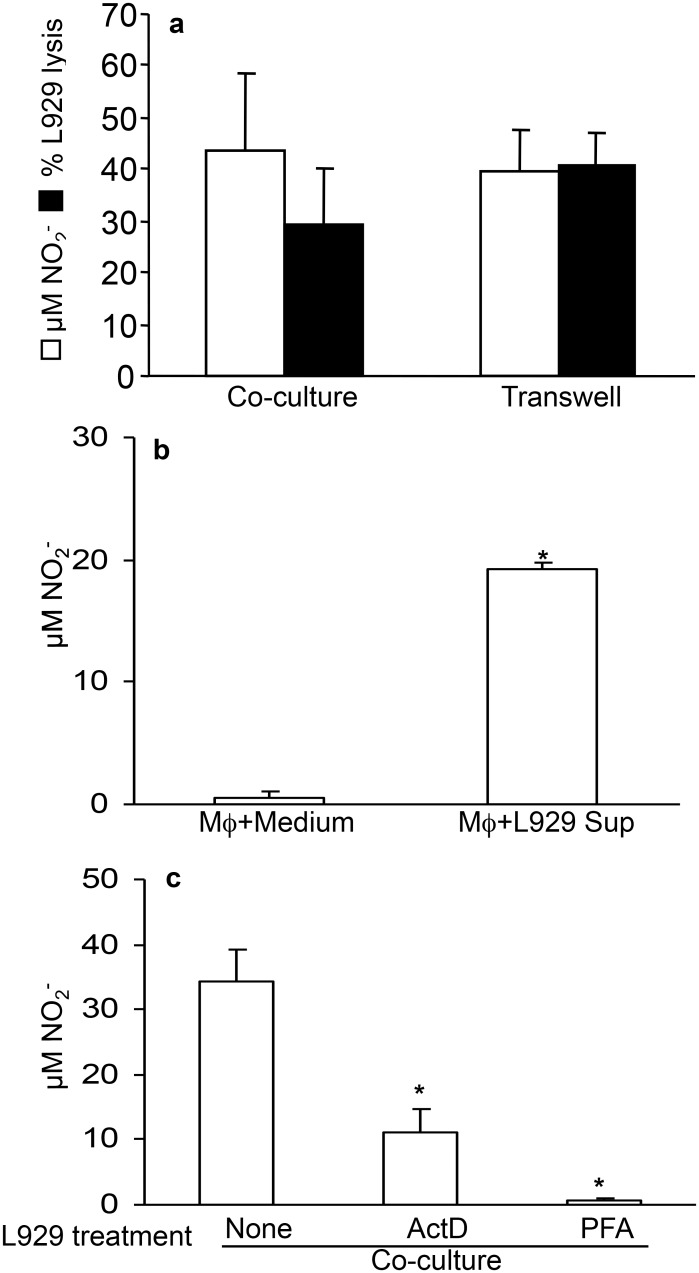
NO production and cytotoxic activity by macrophages co-cultured with L929 is independent on cellular contact. **(a)** Resident macrophages were cultured separated from L929 cells by a cell-dense membrane with 0.4 μM pore size (Transwell). **(b)** Resident macrophages were cultured with L929 cells supernatant. C. Resident macrophages were co-cultured with L929 cells treated with 2μg/mL ActD or fixed with 1% PFA. After 48h of each of these cultures, the nitrite concentration was determined by Griess Reaction and macrophage cytotoxicity by crystal violet method. One representative experiment out of three is shown. Data represent the means ± SD (n = 5), *p<0.05.

To determine whether cell-to-cell contact is required for NO production and tumor cytotoxicity we established co-cultures using a transwell system that separated L929 cells from resident macrophages by a cell-impermeable membrane. We found that cell-to-cell contact was dispensable for NO production and L929 cell lysis as these events persisted in transwell cultures (Fig. 2a). These results indicated that the cross talk between macrophages and tumor cells was mediated by soluble factor(s). Indeed, addition of L929 supernatant to resident macrophage cultures significantly induced NO production (Fig. 2b). This mechanism was largely dependent on RNA transcription and viable L929 cells, as ActD treatment inhibited this effect and treatment with paraformaldehyde (PFA) completely abolished the effect of L929 supernatant on macrophages (Fig. 2c). Based on these data, we concluded that L929 cells induction of NO production in macrophages was contact independent and exerted by soluble factors that required mRNA transcription.

### 3. TNFα is not involved in L929 cell lysis by macrophages

**Fig 3:**
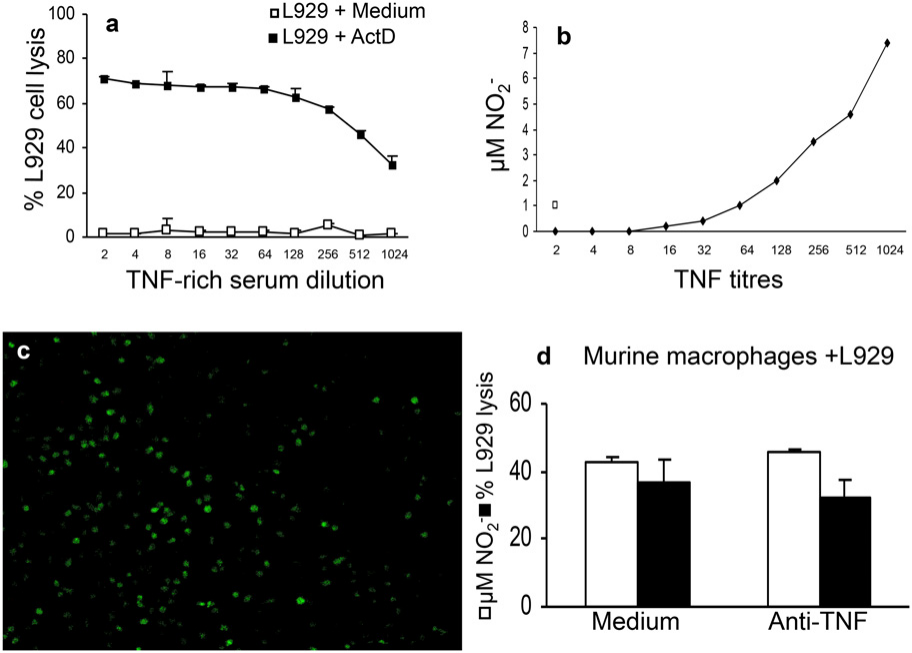
TNFα effect on ActD-treated or non-treated L929 cells. A and B. Serial dilutions of TNFα enriched mouse serum^§^ were added to L929 cells (3.5x10^4^/well) after 24 hours pre-incubation in regular complete medium. **(a)** Half of the cells in the plate were also treated with 2 μg/ml ActD. After 24 hours, the cellular viability was determined by crystal violet method. One representative experiment out of three is shown. Data represent the means ± SD of quadruplicates. **(b)** Nitrite concentration in TNFα treated L929 cultures, measured by Griess Reaction. **(c)** L929 cell (3.5x10^4^/well) cultures in the presence of maximum concentration of TNFα rich serum for 48h^§^. 12.5 μM DAF (green) was added to the cultures to be incorporated in NO producing cells and detected under an epifluorescence microscope. **(d)** Resident macrophages (2x10^5^/well) were co-cultured with L929 cells (3.5x10^4^/well) in the presence or absence of 20μg/mL anti-TNFα. After 48h, the concentration of nitrite was determined by Griess Reaction and macrophage cytotoxicity by crystal violet method. ^§^TNFα rich mouse serum was obtained by infecting mice with 2.5x10^7^ *Mycobacterium bovis* bacilli, followed by treatment with 35 μg LPS 15 days post injection. Mice were euthanized 90 min later and blood harvested for serum preparation [28].

ActD treated L929 cells became extremely susceptible to TNFα and as such it is used as a biological test to measure TNFα activity [29,30]. Indeed, the susceptibility of L929 cells to TNFα only became apparent when L929 cells were treated with ActD (Fig. 3a). Interestingly, at high concentration of TNFα, L929 cells (not treated with ActD) produced low levels of NO as revealed by Griess reagent and DAF staining, indicating that L929 cells are able to produce NO (Fig. 3b). However, at these concentrations of NO there was no change in L929 cell viability (Fig. 3a, 3c). Having established that in our conditions TNFα do not induce L929 lysis, we investigated whether TNFα is involved in tumoricidal activity in our co-cultures of L929 cells and murine macrophages using anti-TNFα antibodies. Blocking TNFα with a neutralizing antibody had no effect on NO production or L929 cell lysis (Fig. 1d).

### 4. iNOS activity is important for maximal NO production in co-cultures of L929 cells and macrophages

**Fig 4:**
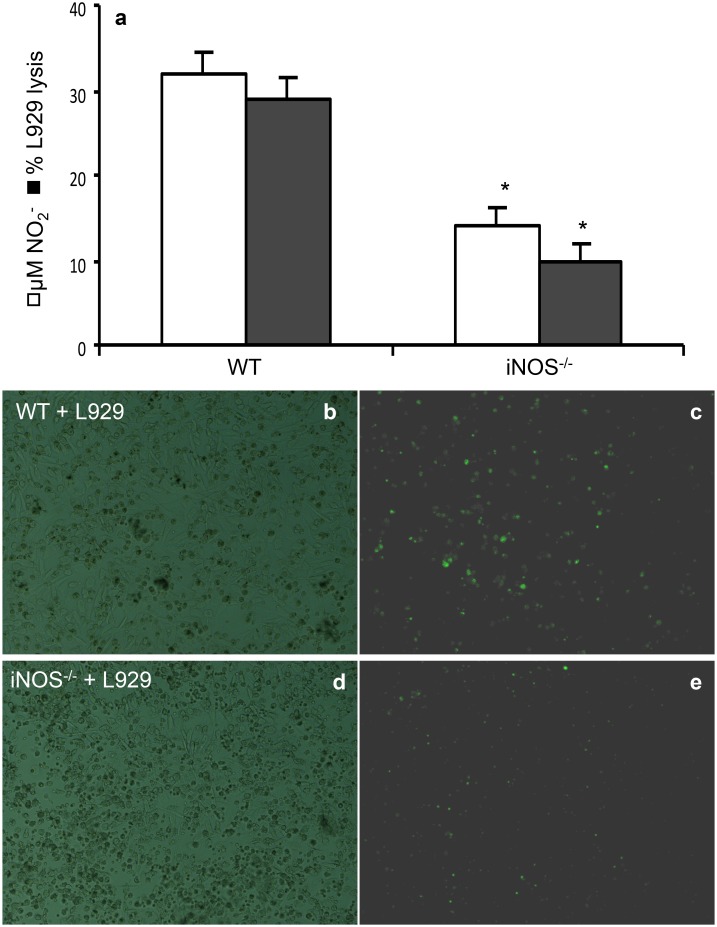
Nitric oxide production and L929 cells lysis by resident macrophages. **(a)** Resident macrophages (2x10^5^/well) from C57Bl/6 or C57Bl/6 iNOS KO mice were co-cultured with L929 cells (3.5x10^4^/well) for 48 h. Nitrite levels were determined by Griess reaction and macrophage cytotoxicity by crystal violet method. One representative experiment out of three is shown. Data represent the means ± SD (n = 5). **(c,d)** Detection of NO producing cells using DAF. 12.5 μM DAF was added to cultures to be incorporated into NO producing cells from C57Black/6 mice **(c,d)** and from iNOS deficient mice **(e,f)**. **(b)** and **(d)** are bright field acquired images to illustrate the presence of a cell monolayer.

High concentrations of NO are produced upon activation of iNOS [31]. Therefore; we determined the role of iNOS in NO production and L929 cell lysis, using iNOS-deficient macrophages. We found a significant reduction in NO concentration and DAF staining of co-cultures of iNOS KO macrophages with L929 cells compared to co-cultures with control wild type (WT) macrophages (Fig. 4a-e). As expected, L929 cells lysis also decreased (Fig. 4a). Notably, although NO production was decreased in co-cultures with iNOS-deficient macrophages as revealed by Griess reaction and DAF staining, it was not abolished (Fig. 4a, 4e); suggesting that residual NO production was probably derived from L929 cells. These results suggest that macrophages and L929 cells stimulate each other for NO production and in the absence of iNOS expression in macrophages, there is still residual NO production by L929 cells.

### 5. IRF-1 but not MyD88 adaptor molecule is essential for NO production by macrophage-L929 cells co-cultures

**Fig 5:**
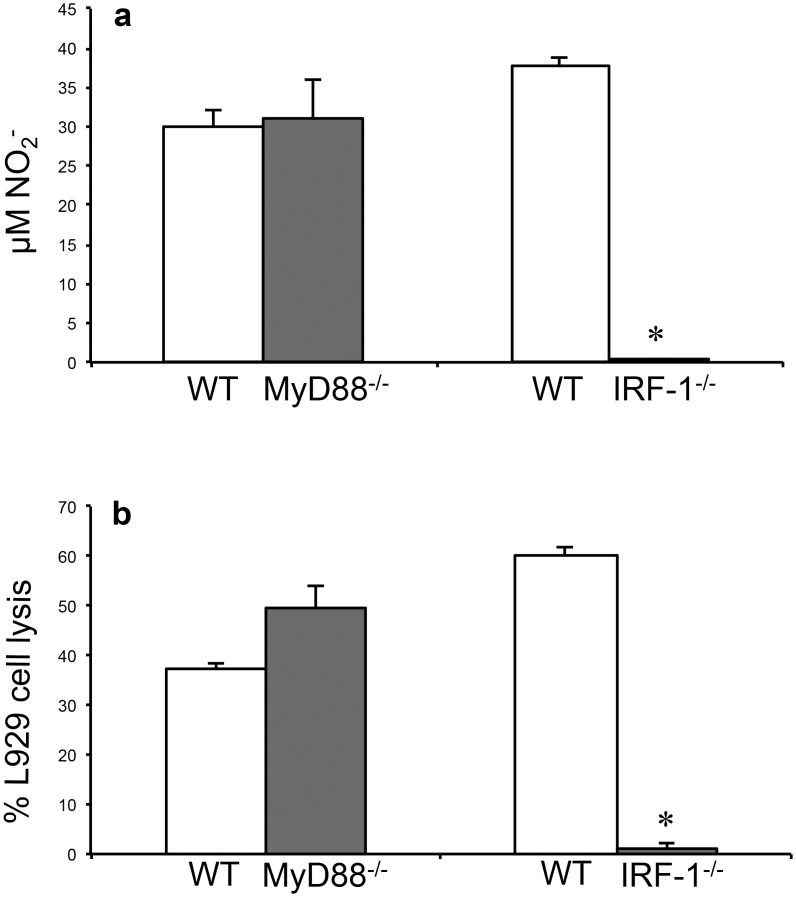
Nitric oxide production and L929 cells lysis by resident macrophages from MyD88 and IRF-1 deficient mice. Resident macrophages (2x10^5^/well) from MyD88 deficient (MyD88-/-) or IRF-1 deficient (IRF-1-/-) mice were co-cultured with L929 cells (3.5x10^4^/well) for 48 h. **(a)** Nitrite levels were determined by Griess Reaction. **(b)** Macrophage cytotoxicity was determined by crystal violet method. One representative experiment out of three is shown. Data represent the means ± SD (n = 5/group).

**Fig 6:**
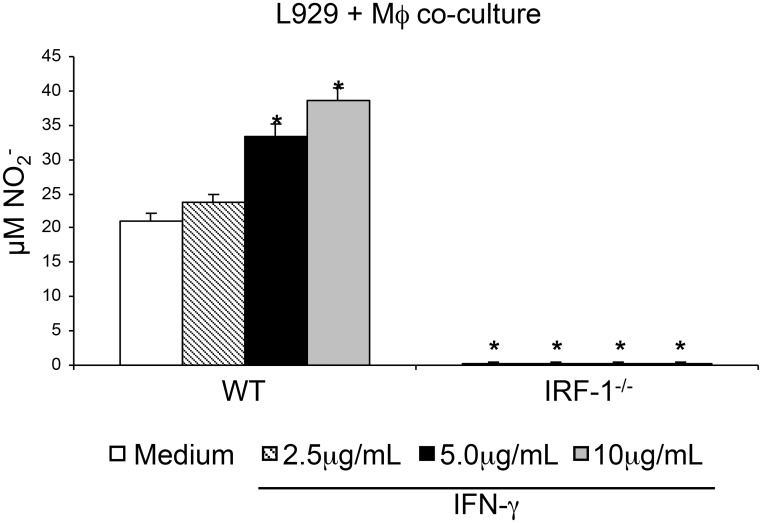
Nitric oxide production by resident macrophages is dependent on IRF-1. Resident macrophages (2x10^5^/well) from wild type or IRF-1 deficient mice (IRF-1-/-) were incubated with L929 cells (3.5x10^4^/well) in the presence of the indicated concentrations of IFNγ. After 48 hours of incubation, cell supernatants were used for nitrite concentration determination by Reaction. Data represent the means ± SD (n = 5/group).

Since it was shown that IRF-1 activity is essential for iNOS expression in mice [12] and that MyD88 molecule, an important adaptor molecule in Toll-Interleukin-1 receptor signalling, also participate in the induction of NO production [6] we used co-cultures of L929 cells with IRF-1 or MyD88 deficient macrophages. MyD88 deficiency did not influence NO production (Fig. 5a) or L929 cell lysis (Fig. 5b). In contrast, IRF-1 was essential for NO production and L929 cell lysis (Fig. 5a-b). These results suggest that the crosstalk between macrophage and L929 cells is essential for these cells to produce NO and entirely dependent on macrophage IRF-1. Since IRF-1 is one of the targets of IFNγ signalling [9] we treated macrophage/L929 co-cultures with IFNγ to induce NO production. Indeed IFNγ induced NO production in a dose dependent manner in co-cultures of L929 cells with WT macrophages. However, NO production was completely abolished in co-cultures with IRF-1 deficient macrophages (Fig. 6).

### 6. Induction NO production by peritoneal macrophages from mice inoculated with L929 cells

**Fig 7:**
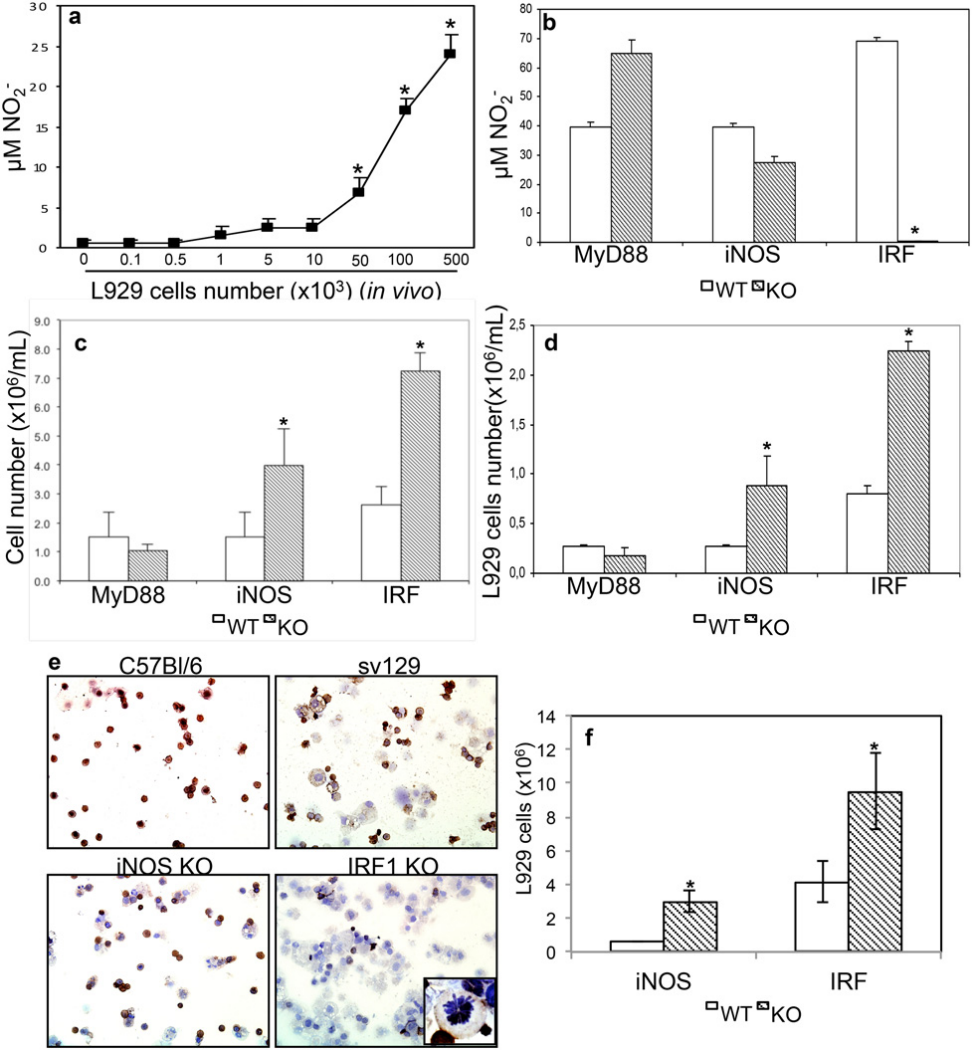
Control of L929 cell growth *in vivo* requires iNOS and IRF-1. **(a)** Indicated numbers of tumor cells were injected i.p. in wild type mice. Seven days later, the peritoneal cells were harvested and 2x10^5^/well seeded. After 48h of *ex vivo* culture, nitrite concentration was determined by Griess Reaction. **(b-d)** L929 tumor cells, 10^5^/mouse, were inoculated into the peritoneal cavity of wild type, MyD88 deficient (MyD88), iNOS deficient (iNOS) and IRF-1 deficient (IRF) mice. **(c)** Quantification of total number of cells from the peritoneal lavage determined by crystal violet staining. **(d)** Quantification of L929 cells in the peritoneal lavage by crystal violet staining, among all cells, L929 were identified by size. One representative experiment out of two is shown. Data represent the means ± SD (n = 3/L929 cells concentration), *p<0.05. **(e)** Immunocytochemistry for detection of CD45 cells in the peritoneal cells harvested from mice injected with L929 cells. Cells fixed in a glass slide using a cytospin centrifuge were incubated with biotinylated anti-CD45, which was detected with spreptavidin conjugated with horseradish peroxidase, which reacts with DAB. Cells were counterstained with hematoxylin. The inset in 1000 X magnification shows the detail of a CD45- cell in mitosis. In all other figures, magnification power was 100X. Experiment representative of two independent ones. **(f)** Quantification of CD45+ and CD45- cells, where we assume CD45- cells in the peritoneal cavity corresponds to L929 cells. Data corresponds to the average of triplicates from two independent experiments.

Concluding that L929 cells induce NO production by macrophages *in vitro*, we next tested if this phenomenon would also occur *in vivo*. We inoculated different concentrations of L929 cells in C57BL/6 mice peritoneal cavities and harvested cells 7 days later. We observed that NO production by peritoneal macrophages increased in direct proportion with the number of L929 cells inoculated in the peritoneal cavity (Fig. 7a). Therefore, L929 cells could stimulate peritoneal macrophages *in vivo* for NO production.

### 7. In vivo NO production and L929 cell lysis is dependent on IRF-1 expression

After determining that L929 inoculated in the peritoneal cavity could stimulate resident macrophages, we aimed to determine the molecular mechanism responsible for this effect. We inoculated L929 cells in the peritoneal cavity of MyD88, iNOS, and IRF-1 deficient mice and 7 days later, harvested peritoneal cells to quantify NO production. As expected, MyD88 deficiency had no effect on NO production, these cells even showed an increase in NO concentration in their supernatant. In cells from iNOS deficient mice, we observed a decrease in NO production, indicating the iNOS contribute, but it is not critical for NO production. However, as observed before, IRF-1 was essential for NO production, since NO production was completely abolished in cells from IRF-1 deficient mice (Fig. 7b). Next, we quantified L929 cell growth by total and differential cell counts using morphologic criteria, which in the case of L929 cells was the size (larger than leukocytes), cytoplasm/nuclei ratio (higher than in leukocytes) and mitosis figures. We found that the total number of cells harvested from the peritoneal cavity of these mice 7 days after inoculation was significantly higher in iNOS deficient mice and even higher in IRF-1 deficient mice when compared with wild type (WT) or MyD88 deficient mice (Fig. 7c). Increase in L929 cell numbers was similar to the pattern observed for the total number of cells (Fig. 7d). We further confirmed these results by staining peritoneal total cell suspensions with anti-CD45 antibody and estimated the number of negatively stained cells, correspondent to L929 cells, either by immunohistochemistry (Fig. 7e-f) or by flow cytometry (S2 Fig.). We not only observed an increase in L929 cells in iNOS and IRF-1 deficient mice, we could also see L929 dividing cells in the peritoneal cavity of IRF-1 deficient mice, indicating that these mice could not control L929 cells growth.

## Discussion

The main finding of the present work indicates that resident macrophages co-cultured with L929 tumor cells, without addition of any exogenous cytokine, are able to produce NO and kill L929 tumor cells in a NO dependent- cell contact-independent manner. Indeed, we found that resident macrophages incubated with conditioned medium of L929 cells produced NO. However, PFA-fixed L929 cells did not stimulate NO production indicating that viable L929 tumor cells are required for NO production. By comparing our findings with previous reports we want to highlight some important differences. First, we found that exogenous stimulants were not required for NO production while other reports showed that NO production is only observed after addition of IFNγ or IFNγ plus TNFα to co-cultures of RAW 264.7 macrophages with L929 cells [19]. Similarly, Zembala and collaborators [32] showed that human monocytes produced NO in co-cultures with L929 cells only in the presence of exogenous cytokines while we found a significant NO production in co-cultures of resident macrophages with L929 cells. Second, Isobe and Nakashima and Nozaki and collaborators demonstrated an essential role of cell-to-cell contact for NO production while in our model cell-to-cell contact was not important [21,22]. Third, Calorini and collaborators described that L929 fibrosarcoma stimulated NO secretion only in co-cultures with inflammatory macrophages but not with resident macrophages [3]. Finally, there is data showing that activated macrophages could lyse L929 cells in a TNFα dependent, NO independent mechanism [33,34]. It is important to note that in these experiments the period of incubation of co-cultures was 18 hours, and at that time we could not detect significant L929 lyses that only occurred after 24 hours of co-cultures.

Our evidence of a cytotoxic mechanism dependent on NO production comes from the experiments where we inhibited NO production or generated NO in the medium through a donor. In contrast, we could not reveal a role for TNFα involvement since blocking of TNFα with neutralizing antibody had no effect on L929 lysis, either using mouse resident macrophages. The differences between our results and those reported above are still elusive, but might be related to the L929 cell line, macrophage source and handling and different protocols used. However, our L929 cell line when treated with ActD became extremely sensitive to TNFα-mediated cytotoxicity that was blocked by anti-TNFα treatment confirming previous reports about this cell line [35,36].

TNFα and NO, as well as other mediators secreted by tumor inflammatory infiltrate, mainly macrophages and myeloid cells, have been reported to exert contrasting effects on tumor cells. Cytotoxic effects of TNFα and NO on tumor cells are well established [37,34,38]. However, chronic inflammatory signalling can stimulate tumor progression through the inhibition of T cell activity via NO, alteration in tumor cell signalling and accumulation of mutations [39,40,41,42]. Some tumor cells also generate inflammatory mediators, resulting in a complex tumor microenvironment that is fundamental for determining tumor fate [43,44].

In line with our observations with TNFα treatment and NO production, it was shown that L929 tumor cells treated with a combination of IFNγ and IL-1 displayed a significant increase in NO production and cell death indicating that L929 cells do produce NO that, in turn, depending on the concentration, has an autocrine cytotoxic effect [17]. Interestingly, using macrophages from iNOS deficient mice in the co-cultures with L929 cells, we verified that although low, NO production persisted, suggesting that, in our experimental conditions, L929 cells might also release this metabolite. Indeed, we found that L929 cells positive for a dye that marks NO production (Fig. 3d). The fact that besides producing NO, L929 tumor cells stimulated resident macrophages to produce this metabolite, led us to investigate the molecular mechanisms involved in this cross-activation. L929 cells secrete cytokines as M-CSF, which can induce differentiation of bone marrow cells into macrophages [45]. Neutralizing of M-CSF had no effect on NO production in resident macrophages in co-culture with L929 cells (data not shown). Indeed, M-CSF signals mainly through PI3K and MAPK, possibly without involvement of the IRF-1 pathway. However, L929 cells also secrete type I IFNs that are known to induce iNOS activation in different cell types [46,9]. We tested NO production in co-cultures of L929 cells with resident macrophages from type I and type II IFN receptors. In both cases, we observed a partial reduction of NO production compared to cultures with WT macrophages (data not shown). This result indicates that both type I or II IFNs are involved in NO production. The pathways triggered by these cytokine receptors activate STAT1 and/or STAT2 leading, among other factors, to activation of IRF-1 [47,48,49,50]. IRF-1 was originally identified as a nuclear factor that bound specifically to the IFNβ promoter and its cDNA was subsequently cloned from murine L929 fibroblasts [10]. IRF-1, together with NF-κB and STAT1 transcription factors, may bind to the promoter of iNOS gene, inducing NO production in macrophages. IRF-1 is associated to the IFNs-related mechanism of activation of iNOS in macrophages [11,12,51]. Therefore, our next step was to investigate the participation of theses molecules in the cross talking between these two cell populations.

Tumor cells may generate DAMPs, TLR ligands, activating the TLR/MyD88/NFκB pathway [52]. However, as shown MyD88 deficient macrophages were still able to produce NO and kill L929 cells in co-cultures, thus emphasizing the independence of the MyD88 triggered signalling in NO production and macrophage cytotoxicity. On the other hand, the NO production in the co-cultures of resident macrophages from IRF-1 deficient mice with L929 was totally abrogated in both cell populations. Besides, the addition of IFNγ in the co-culture did not induce detectable amounts of NO when the macrophages were IRF-1 deficient, suggesting that in these conditions, IFNγ by itself was not able to induce NO production neither by resident macrophages nor by L929 tumor cells. These results clearly show that the crosstalk between both cell populations is strictly dependent on IRF-1 and independent on MyD88 molecule in macrophages. Moreover, our results are in line with a report showing that mildly acidic pH inhibited NO production, through inhibition of IRF-1 expression, protecting cells from death [53].

The essential role of IRF-1 and the substantial role of iNOS in macrophage cytotoxic activity lead us to investigate the role of these molecules in tumor growth *in vivo*. Notably, we found that IRF-1 deficient mice, when inoculated with L929 tumor cells, were not able to produce NO and were extremely susceptible to tumor cells growth when compared with WT mice. We found that the role of iNOS was not as important as IRF-1, since iNOS deficient mice were more susceptible to tumor growth when compared with WT mice, but in comparison with IRF-1 deficient mice iNOS deficient mice were less permissive to tumor growth.

**Fig 8:**
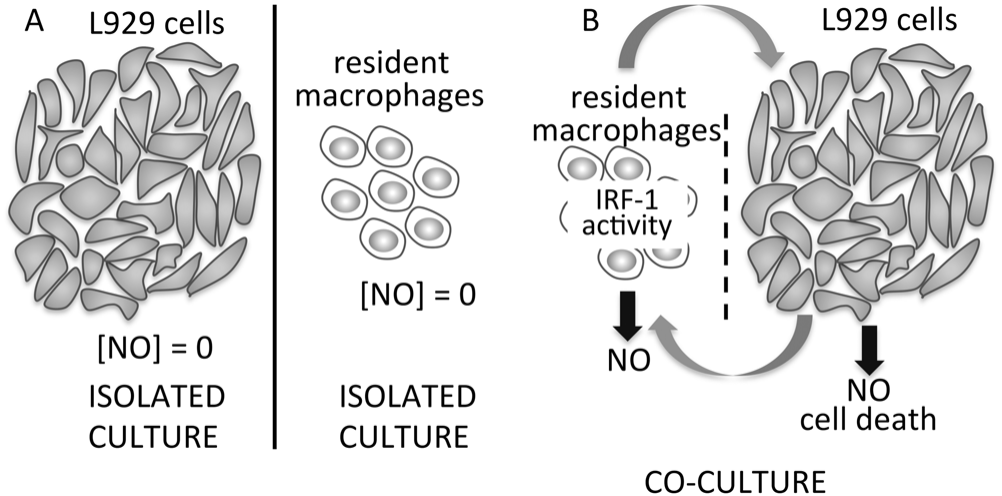
Schematic representation of NO production in cultures and co-cultures of macrophages and L929 cells. A. In L929 cells and macrophages isolated cultures, we did not detect NO production within the sensitivity of the tests we used. B. In co-cultures with or without cell-cell contact (dashed line represents insert separating cell types) NO production increased in both macrophages and L929 cells, leading to L929 cell death. IRF-1 activity in macrophages was an essential factor in promotion of NO synthesis and cytotoxicity.

In conclusion, we have clearly shown a cross talk between macrophages and L929 tumor cells, in the absence of any exogenous cytokines, which stimulates murine resident macrophages to produce NO and became cytotoxic by an IRF-1 dependent and contact-independent route (Fig. 8). In addition we showed that type I and type II IFNs are partially involved in the macrophage stimulation by L929 tumor cell. Most importantly, we have shown that the cross-stimulation of L929 tumor cell and resident macrophage is strictly dependent on IRF-1. In the absence of this key molecule the macrophages were unable to produce NO and to kill the tumor cells *in vitro* and *in vivo* the mice became extremely permissive to tumor proliferation.

Our work shows a cross-talk between macrophages and L929 cells where IRF-1 and NO production work against tumor cells by inducing L929 cell death *in vitro* and control of tumor growth *in vivo*.

## Supporting Information

S1 Fig Crystal violet staining and NO production controls in tumor cells and macrophage cultures. a. 3.5x10^4^ L929 cells/well or 2x10^5^ peritoneal cells/well were seeded in flat bottom 96 wells plates; 72 hours later, cells were washed, fixed, and stained with 0.1% crystal violet in 6% acetic acid. After air-drying, the stain was solubilized in 100 μl methanol and final product O.D. measured at 630 nm. The graph shows percentage of staining, where L929 optical density was considered 100%. b. NO production in 72 hour cultures of L929 cells (initially seeded at 3.5x10^4^ cells/well) or macrophages (initially seeded at 2x10^5^ cells/well) or co-cultures, established by seeding macrophages over a L929 monolayer (initially seeded at 3.5x10^4^ cells/well). Nitrite concentration was determined by comparison of O.D. at 540 nm with a standard curve.
(TIF)

S2 Fig Flow cytometry analyses of peritoneal cells from WT, IRF1-/- and iNOS-/- mice injected with L929 cells. This experiment allowed us to identify and differentiate L929 cells from leukocytes present in the peritoneal cavity.
(TIF)
