# Supplementary figures and images for: Interferon Regulatory Factor (IRF)-1 Is a Master Regulator of the Cross Talk between Macrophages and L929 Fibrosarcoma Cells for Nitric Oxide Dependent Tumoricidal Activity
Source: PLoS One. 2015 Feb 6;10(2):e0117782. doi: 10.1371/journal.pone.0117782 (PMC4449231; doi:10.1371/journal.pone.0117782)

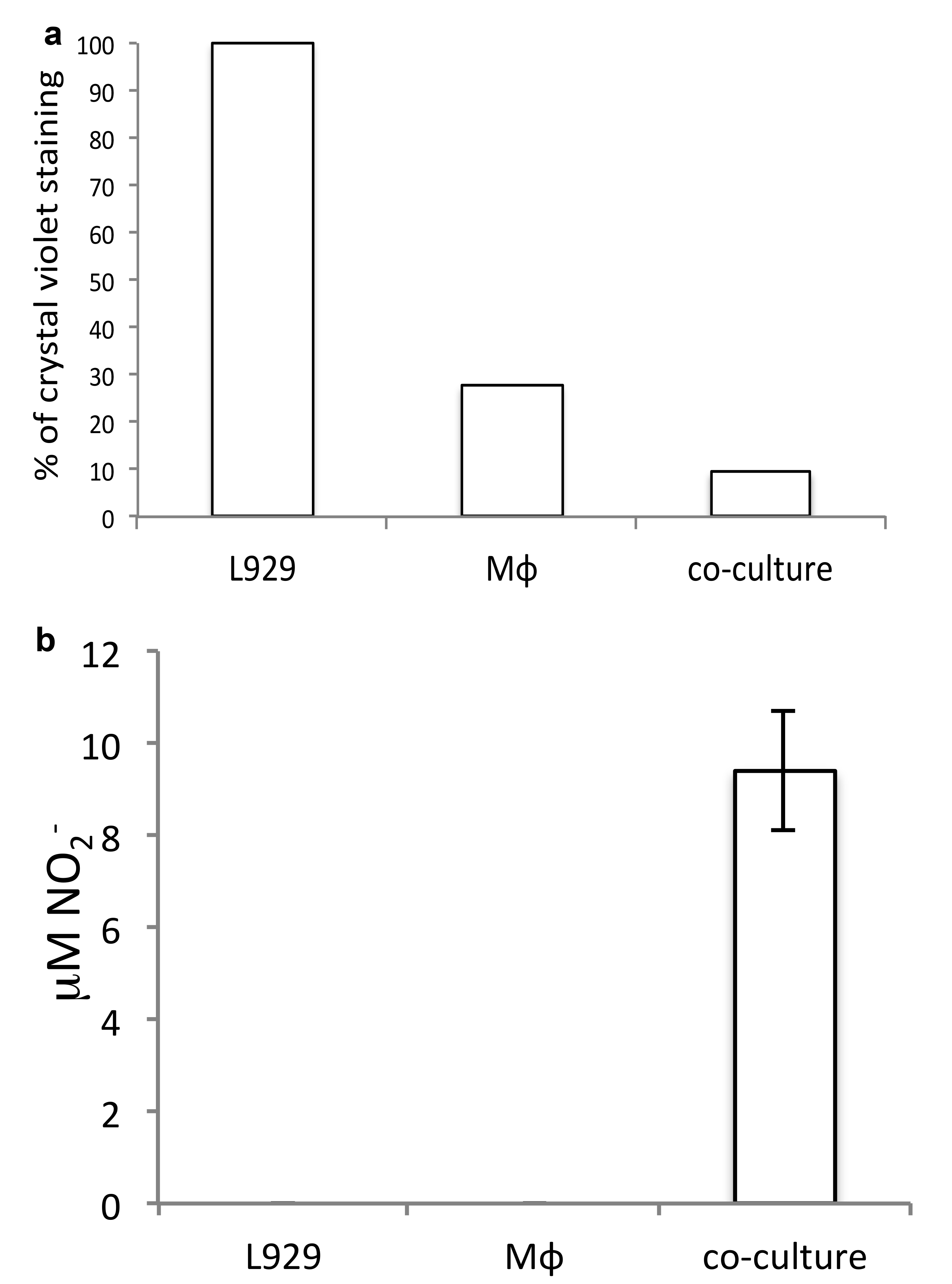

Supplement: S1 Fig — a. 3.5x10^4^ L929 cells/well or 2x10^5^ peritoneal cells/well were seeded in flat bottom 96 wells plates; 72 hours later, cells were washed, fixed, and stained with 0.1% crystal violet in 6% acetic acid. After air-drying, the stain was solubilized in 100 μl methanol and final product O.D. measured at 630 nm. The graph shows percentage of staining, where L929 optical density was considered 100%. b. NO production in 72 hour cultures of L929 cells (initially seeded at 3.5x10^4^ cells/well) or macrophages (initially seeded at 2x10^5^ cells/well) or co-cultures, established by seeding macrophages over a L929 monolayer (initially seeded at 3.5x10^4^ cells/well). Nitrite concentration was determined by comparison of O.D. at 540 nm with a standard curve. (TIF) [file pone.0117782.s001.tif]

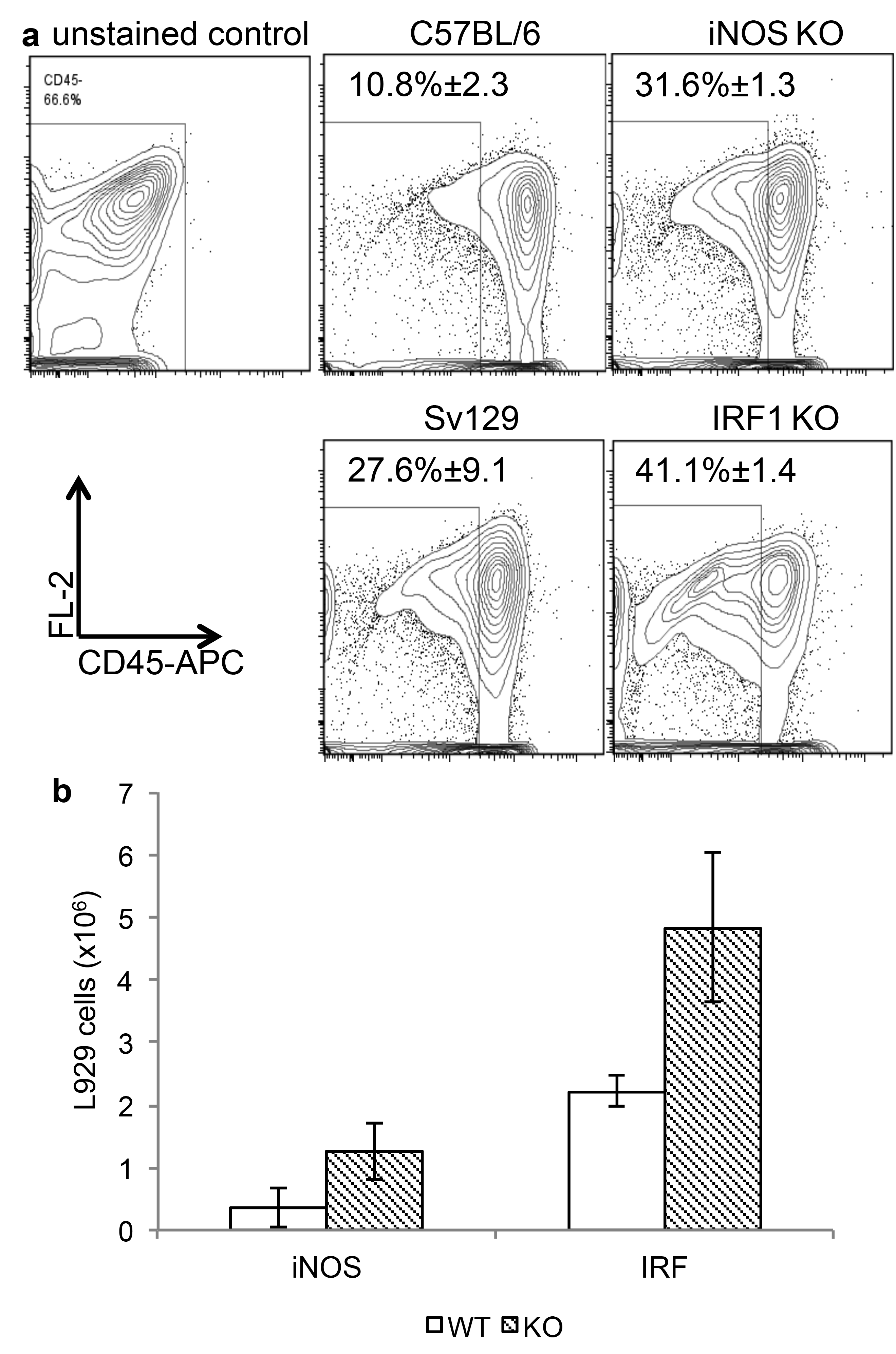

Supplement: S2 Fig — This experiment allowed us to identify and differentiate L929 cells from leukocytes present in the peritoneal cavity. (TIF) [file pone.0117782.s002.tif]
